# Disseminated ovarian granulosa cell tumor after laparoscopic surgery: Two case reports

**DOI:** 10.1097/MD.0000000000025176

**Published:** 2021-04-16

**Authors:** Man-Hua Cui, Xi-Wen Zhang, Li-Ping Zhao, Shu-Yan Liu, Yan Jia

**Affiliations:** Department of Gynecology and Obstetrics, The Second Hospital of Jilin University, Changchun, Jilin Province, China.

**Keywords:** granulosa cell tumors, laparoscopic, ovary, power morcellation

## Abstract

**Rationale:**

Granulosa cell tumors (GCT) have an incidence of 0.6 to 0.8/100,000. Short-term relapsed ovarian GCT is extremely rare. Herein, this report aims to present 2 rare cases of disseminated ovarian GCT and analyze the causes of recurrence.

**Patient concerns:**

The 2 patients presented with abdominal pain.

**Diagnosis:**

Both the patients were diagnosed with relapsed ovarian GCT (IIIc stage) in the adult type.

**Interventions:**

The 2 patients had a medical history of surgery for ovarian GCT by using laparoscopic with power morcellators (LPM). They experienced relapsed ovarian GCT postoperatively. Subsequently, they received a repeated operation through a laparotomy approach. Numerous malignant metastasis neoplasms were detected at the port-sites. Then, tumor resection was performed.

**Outcomes:**

The postoperative pathologies of both case 1 and case 2 reported ovarian GCT (IIIc stage) in adult type. The 2 patients presented disease-free survival for more than 33 months follow-up period.

**Lessons:**

The application of LPM may be a risk factor of disseminated ovarian GCT. However, laparoscopic surgery is still an optimal treatment strategy for ovarian tumors. Besides, gynecologists should comply with the tumor-free principle during surgery.

## 1 Introduction

Granulosa cell tumors (GCT) derive from the ovarian mesenchyme and sex cords.^[1–4]^ It accounts for approximately 70% of all sexual stromal tumors and 5% to 8% of all ovarian tumors.^[1–4]^ The endocrine function of GCT can lead to early endocrine-related clinical symptoms, which is beneficial to the early diagnosis of this disease. Levin et al^[5]^ have found that the probability of GCT patients being diagnosed in the I, II, III, and IV stage was 74% to 95%, 5.1% to 11%, 0.8% to 10%, and 0.5% to 8.6%, respectively.

GCT is divided into juvenile granulosa cell tumors (JGCT) and adult granulosa cell tumors (AGCT).^[6–8]^ Among all GCT, AGCT accounts for about 90% to 97%, and JGCT accounts for about 3% to 10%.^[6–8]^ The main clinical manifestations of AGCT include irregular vaginal bleeding (45%),^[9]^ abdominal pain, distension (10–20%),^[9]^ and other manifestations, such as endometrial thickening, pelvic mass, and ascites. AGCT is common in perimenopausal women and has a median onset age of 50 to 55 years.^[10]^ Unilateral AGCT occurs in 95% of cases, and it occurs more often on the right side (55.9%).^[2,4,7,8]^

GCT is common in gynecology. However, short-term disease recrudescence after laparoscopic with power morcellators (LPM) is relatively infrequent. Therefore, we reported 2 rare relapsed AGCTs and evaluated the reasons for tumor implantation and metastasis.

## 2 Case report

This case report was approved by the institutional review board of the Jilin University Second Hospital. Informed written consent was obtained from the patient for publication of this case report and accompanying images.

### 2.1 Case 1

**Figure 1:**
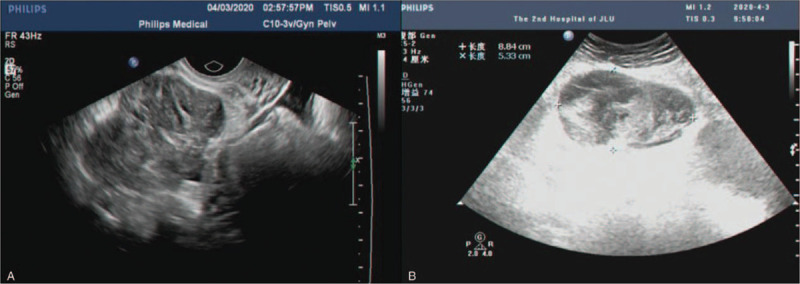
Abdominal ultrasound results of case 1. (A) The ultrasound suggested a 5.9 cm × 4.2 cm heterogeneous hypoechoic between the uterus's anterior wall and the posterior wall of the bladder. (B) The ultrasound indicated multiple hypoechoic nodules in abdominal. Moreover, a big mass was found below the periumbilical abdominal wall.

**Figure 2:**
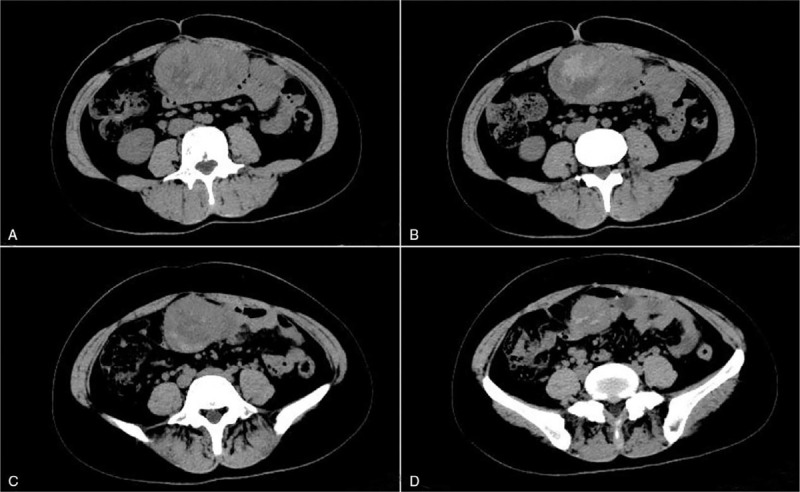
Abdominal computed tomography of case 1. (A and B) A 5.8 cm × 9.0 cm mass was found at the umbilicus level. (C and D) A 3.5 cm × 4.5 cm soft tissue mass was observed at 5 cm below the umbilicus and at the outer edge of the left rectus abdominis.

A 35-year-old female patient came to our outpatient clinic office complaining of lower abdominal pain for 15 days. She was married, had 4 pregnancies and 2 cesarean deliveries. Fifteen months before, the patient underwent laparoscopic surgery to remove the right accessory for the right AGCT in another hospital. Chemotherapy was not performed but the patient was under a regular follow-up. Fifteen days before, the patient developed abdominal pain. Abdominal color doppler ultrasound and total abdominal CT scan revealed multiple pelvic and abdominal masses (Figs. 1 and 2). Subsequently, she received a second operation through an open abdominal approach, including total hysterectomy, left salpingo-oophorectomy, pelvic lymphadenectomy, significant omentum resection, and appendectomy, and tumor cell reduction were performed.

**Figure 3:**
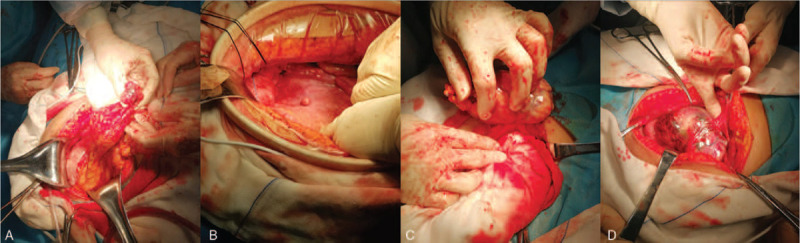
Clinical pictures of case 1. (A) The mass was found in the left abdominal wall. (B) An isolated lesion implanted in the peritoneum was detected. (C and D) The mass was embedded in the umbilicus's lower abdominal wall and attached to the abdominal wall, with a 1.5 cm pedicle width.

Intraoperatively, we found 4 disseminated niduses around the laparoscopic port-sites (Fig. 3). Lesion 1 was found at the periumbilical's peritoneal port-site and lesion 2 was detected at the rectus abdominis outer margin's peritoneal site. We also found metastases in the peritoneal turnover adjacent to the bladder (lesion 3) and the left abdominal wall (lesion 4). The characteristics of the lesions are listed in Table 1.

**Table 1:** The basic features of niduses in case 1.

	Position	Size	Single or multiple
Lesion 1	At the periumbilical puncture site of the previous laparoscopic operation, the other side is connected with the greater omentum vessel.	The root pedicle is about 1.5 cm wide	Single
Lesion 2	Bladder reflex peritoneum	–	Multiple plaque shaped lesions
Lesion 3	The lesion is located in the left abdominal wall, surrounded by a large mesh, and its roots are planted in the peritoneum at the puncture site of the outer margin of the rectus abdominis during the previous laparoscopic operation	4.0 cm × 4.0 cm × 3.0 cm and 3.0 cm × 2.0 cm × 2.0 cm, respectively	Two plaque shaped lesions
Lesion 4	Located on the surface of the intestine and peritoneum	–	Multiple

**Figure 4:**
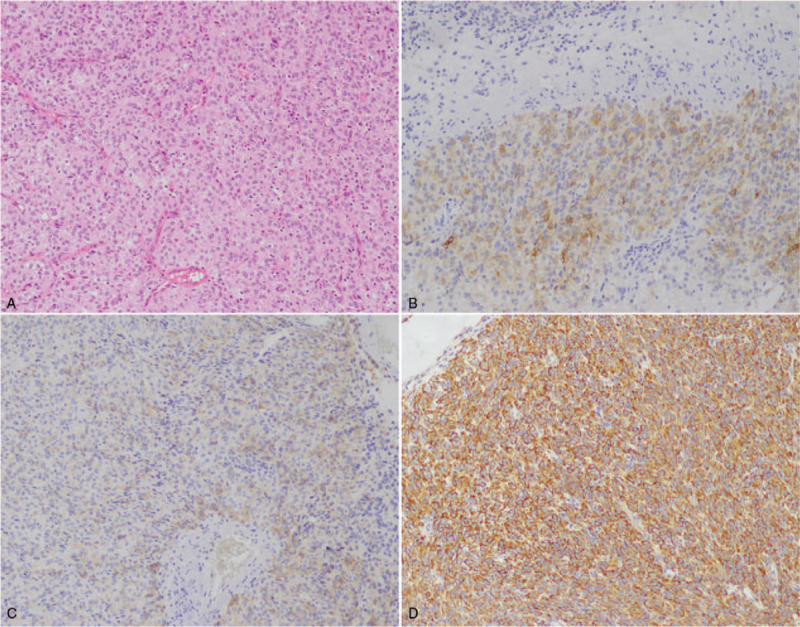
Pathological results of case 1. (A) Haematoxylin-Eosin 100×; (B) Immunohistochemistry, inhibitor bin (local +); (C) Immunohistochemical CD99 (weak +); (D) Immunohistochemical Vimentin (+).

**Figure 5:**
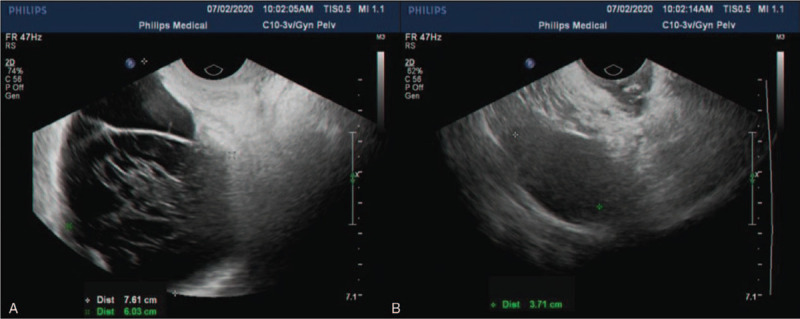
Postoperative gynecological ultrasound of case 1. (A and B) We found a 7.5 cm × 4.9 cm anechoic shape in the left iliac fossa, with an irregular shape, clear boundary, and line-like separation.

Postoperatively, pathological results indicated AGCT (IIIc stage) (Fig. 4). The patient received regular chemotherapy, including paclitaxel (Paclitaxel Injection, 5 mL: 30 mL, Hospira Australia Pty LTD, Australia) and carboplatin (Carboplatin Injection, 10 mL: 100 mg, Qilu Pharmaceutical Co. LTD, China). No tumor reoccurrence was found during 37 months followed up period (Fig. 5).

### 2.2 Case 2

A 51-year-old female patient presented to our hospital with abdominal pain. She was married, pregnant 1, and had a normal birth.

**Figure 6:**
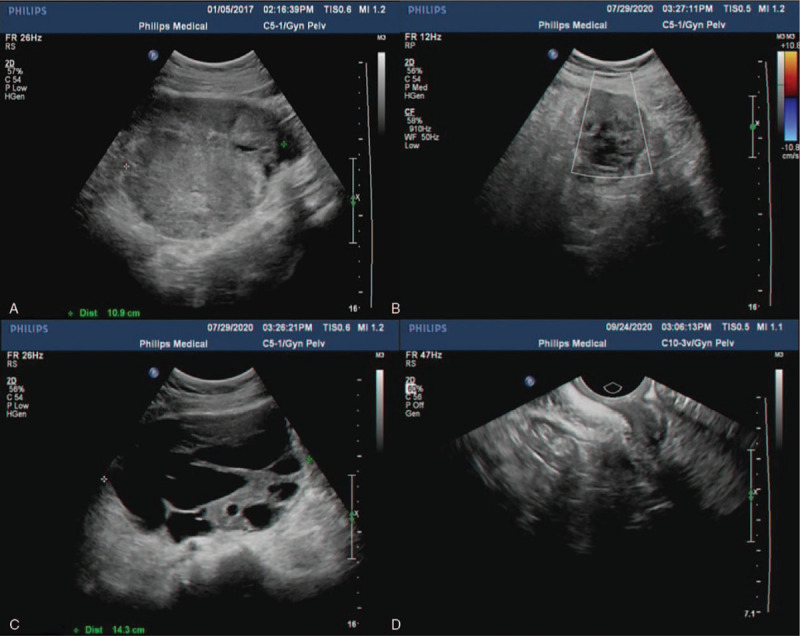
Gynecological ultrasound of Case 2. (A, B, and D) Ultrasound revealed a 6.3 cm × 5.1 cm heterogeneous cystic mass with an irregular shape, unclear boundary, and no blood flow. (C) Above the vaginal stump, we found a 14.3 cm × 8.0 cm cystic mass with regular morphology, no internal blood flow, and multiple compartments.

Forty-four months ago, a mass about 10 cm in diameter was found in her abdomen by ultrasound (Fig. 6A). Then, laparoscopic abdominal exploration was performed for her acute abdominal pain. The surgeon found that the ovary presented 720° torsion intraoperatively, the right ovary and fallopian tube revealed purplish-blue color, and the tumor surface showed multiple ruptures. The pathology of the frozen section during surgery suggested ovarian GCT. Consequently, she underwent a total hysterectomy and bilateral mastectomy by LPM. Postoperatively, she was diagnosed with AGCT (Ic stage). The patients had no discomfort symptoms. The gynecologist advised the patient to have 6 courses of chemotherapy. However, she encountered severe myelosuppression (III degree) after the first chemotherapy. Therefore, she had to stop her follow-up chemotherapy.

**Figure 7:**
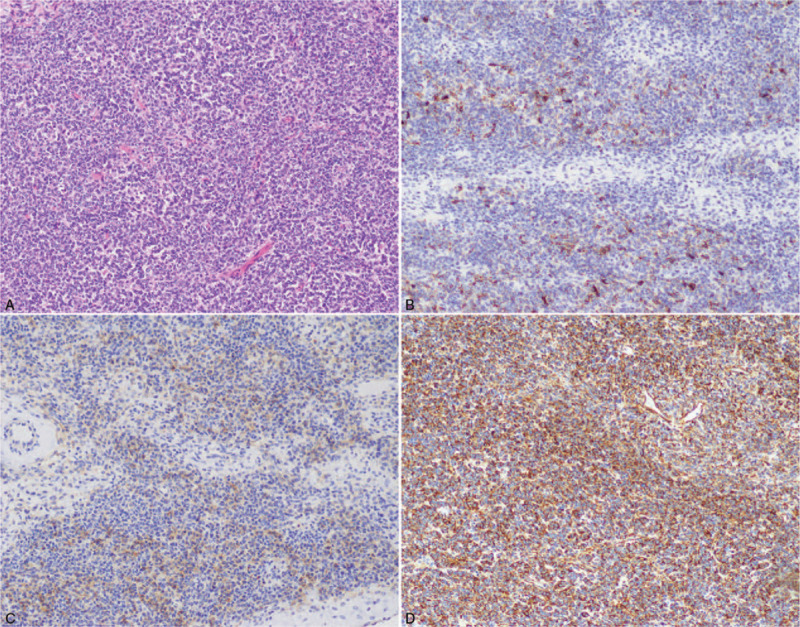
Pathological results of case 2. (A) Haematoxylin-Eosin 100×; (B) Immunohistochemistry, inhibitor bin (+); (C) Immunohistochemical CD99 (+); (D) Immunohistochemical Vimentin (+).

Ten months later, the patient developed abdominal pain and came to our hospital. Abdominal ultrasound suggested a mass about 7 cm in diameter (Fig. 6B and C). She was diagnosed with a recurrence of the malignant tumor. We gave the patient 6 chemotherapy courses with paclitaxel (Paclitaxel Injection, 5 mL: 30 mL, Hospira Australia Pty LTD, Australia) and carboplatin (Carboplatin Injection, 10 mL: 100 mg, Qilu Pharmaceutical Co. LTD, China). Meanwhile, oral targeting drug Eitam (Apatinib Mesylate Tablets, 0.375 g, Jiangsu Hengrui Pharmaceutical Co. LTD, China) was administered for 1 month. However, the reexamination of abdominal ultrasound showed no reduction in mass volume. Thus, we performed open abdominal surgery. A 14.0 cm × 9.0 cm × 8.0 cm cystic mass was detected intraoperatively at the port-site. Besides, multiple metastases of varying sizes were found in the upper abdominal cavity, pelvic peritoneum, spleen, and pancreas. Thus, the pelvic mass, whole spleen, body and tail of the pancreas, and greater omentum were resected. Tumor cell reduction was also performed. The patients were tumor-free during 33 months follow-up visit (Fig. 6D). The postoperatively pathological results indicated the ovarian AGCT (IIIc stage) (Fig. 7A–D). She underwent 3 courses of chemotherapy using paclitaxel (Paclitaxel Injection, 5 mL: 30 mL, Hospira Australia Pty LTD, Australia) and carboplatin (Carboplatin Injection, 10 mL: 100 mg, Qilu Pharmaceutical Co. LTD, China).

## 3 Discussion

GCT originates from the granulosa cells in follicular cells before ovulation. Although AGCT tends to occur in older women than JGCT, discriminating between these subtypes is not based upon patient age, rather on the tumor's histological results.^[11]^ Numerous disseminated neoplasms have been reported as late complications associated with LPM for endometrial cancer, including ectopic leiomyoma,^[12]^ diffuse peritoneal leiomyomatosis,^[13]^ and mature cystic teratoma.^[14,15]^ The pelvis is the most common relapse site, and abdominal and peritoneal disease and retroperitoneal are also occasional metastases site.^[16–18]^ However, disseminated ovarian AGCT after laparoscopy surgery for endometrial cancer has been rarely reported. Consequently, we presented 2 rare recurrent ovarian AGCTs and assessed the causes for neoplasm implantation and metastasis at the port-site.

### 3.1 Surgical treatments for the primary ovarian AGCT

Both laparotomy and laparoscopy can be selected for ovarian AGCT.^[17]^ The gold standard for treating primary ovarian AGCT is complete resection of tumors, uterus, ovaries, and fallopian tubes. Besides, staged peritoneal irrigation, biopsy, and lesser omentum excision are required.^[19]^ Pelvic and para-aortic lymphadenectomy (i.e., surgical removal of lymph nodes) are generally not recommended.^[19]^ However, bulky or suspected cancerous lymph nodes should be removed. Fotopoulou et al reported that the laparoscopic approach was safe and had less morbidity.^[17]^ However, in the current study, 2 patients experienced relapsed ovarian AGCT after LPM. In our opinion, the relapsed ovarian AGCT may be related to the technical shortcomings of LPM.

### 3.2 Surgical treatment for the relapsed ovarian AGCT

To date, no standard surgical approach or treatment protocol was established for the relapsed ovarian AGCT. The treatment of relapsed tumors should aim for optimal surgical debulking whenever possible^[16,18,19]^; this is crucial since the residual tumor can lead to subsequent tumor relapse and affect prognostic.^[4]^ Additionally, relapsed ovarian AGCT patients are likely to benefit from repeated surgical if optimal debulking can be achieved.^[16]^ According to 2 retrospective studies, an optimal debulking with no macroscopic residual tumor can be achieved in most secondary surgery patients.^[17,18]^ Prolonged disease-free survival was reported in a small patient series regardless of the additional risks associated with repeated and extensive surgical procedures.^[20]^ In the present study, we performed optimal surgical debulking for both patients and conducted standardized chemotherapy. During follow-up, 2 patients achieved disease-free survival. We attribute this positive outcome to rational treatment regimens.

### 3.3 Laparoscopic for ovarian AGCT

In 1990, Matvienko and Polishchuk first reported laparoscopy in the treatment of gynecologic diseases.^[21]^ Subsequently, laparoscopy has been widely used in gynecological operations due to its minimally invasive characteristics. Laparoscopic surgical techniques for the treatment of ovarian AGCT require tumor resection completely and surgery duration minimization. In our opinion, laparoscopic can be used to perform a total hysterectomy and adnexectomy with the affected annex side first if the patient does not need to preserve fertility. Then, the removed tissue was placed in a compatible containment system to reduce the exposure time of the tumor in the abdominal cavity. Finally, the tissue can be removed through the vagina along with other excised tissue.

For patients who need to preserve the fertility function, gynecologists could make a small incision in the abdomen to remove the tumor. It also can perform a sizeable single-hole laparoscopy, which is the benefit of removing the tumor. Laparoscopic tumor resection can also be achieved through the vagina.^[22,23]^ The surgeon may also perform the nature orifice transluminal endoscopic surgery^[22,23]^; this approach allows the surgeon to place the laparoscopy through the abdomen and perform an incision in the posterior fornix for tumor removal. However, its safety and efficacy need to be further evaluated in randomized controlled clinical trials.

### 3.4 Laparoscopic power morcellation

In 1993, the LPM was first used in laparoscopic abdominal surgery. In 1995, it was officially approved by the food and drug administration (FDA) for use in laparoscopic surgery. Using LPMs allows for minimally invasive surgical operations, which, when compared to open abdominal surgery, significantly shortens the postoperative recovery period and reduces the risk of infection.^[24–26]^

Case 1 in this report underwent laparoscopic resection of ovarian tumor at the initial onset. Intraoperatively, LPM was used to destroy the tumor envelope without protective measures, resulting in the spread of the granulosa cell tumor fragments. In case 2, the tumor's capsule was ruptured before surgery. In our opinion, power morcellation in the absence of protective measures may induce a malignant spread in the abdominal cavity. Our views on this issue coincide closely with many authors.^[15,26–28]^ Previous studies have reported that using LPM in myomectomy or hysterectomy operations can increase the risk of spreading unsuspected cancer within the abdomen and pelvis.^[15,26–28]^ The CO_2_ gas in the abdomen would be discharged through the port-site, which provides an opportunity for tumor fragments to accumulate at the port-site; this is also the reason why our 2 cases of relapsed tumors were found at the port-site. Consequently, LPM should be used in combination with compatible containment systems to treat ovarian AGCT.^[26]^

## 4 Limitations

We achieved positive disease-free survival results during follow-up in the 2 patients. However, numerous limitations exist in the current study. The follow-up duration of these 2 patients was less than 5 years. Hence, long-term follow-up is needed. Moreover, the sample size in this study was relatively small. Therefore, multicenter, large sample, and randomized controlled trials are required to evaluate the surgical effect of disseminated ovarian AGCT.

## 5 Conclusion

The application of LPM may be a risk factor of relapsed ovarian GCT. However, laparoscopic surgery is still an optimal treatment strategy for ovarian tumors. Besides, gynecologists should comply with the tumor-free principle during surgery.

## Acknowledgments

We would like to thank the patients for their participation in this study.

## Author contributions

**Conceptualization:** Yan Jia, Manhua Cui.

**Data curation:** Xi-Wen Zhang, Li-Ping Zhao, Manhua Cui.

**Formal analysis:** Xi-Wen Zhang, Li-Ping Zhao, Shu-Yan Liu.

**Investigation:** Shu-Yan Liu, Manhua Cui.

**Methodology:** Xi-Wen Zhang, Li-Ping Zhao.

**Project administration:** Yan Jia, Manhua Cui.

**Resources:** Shu-Yan Liu, Manhua Cui.

**Software:** Shu-Yan Liu.

**Supervision:** Manhua Cui.

**Writing – original draft:** Xi-Wen Zhang.

**Writing – review & editing:** Yan Jia, Manhua Cui.
